# Impact of Sodium–Glucose Co-Transporter 2 Inhibitors on Cardiac Protection

**DOI:** 10.3390/ijms22137170

**Published:** 2021-07-02

**Authors:** Victor Chien-Chia Wu, Yan-Rong Li, Chao-Yung Wang

**Affiliations:** 1 Division of Cardiology, Chang Gung Memorial Hospital, Linkou Medical Center, Taoyuan City 33305, Taiwan; victorcwu@hotmail.com; 2 School of Medicine, College of Medicine, Chang Gung University, Taoyuan City 33302, Taiwan; malyr8252@gmail.com; 3 Division of Endocrinology and Metabolism, Department of Internal Medicine, Linkou Chang Gung Memorial Hospital, Taoyuan City 33305, Taiwan; 4 Institute of Cellular and System Medicine, National Health Research Institutes, Zhunan 35053, Taiwan; 5 Department of Medical Science, National Tsing Hua University, Hsinchu 30013, Taiwan

**Keywords:** SGLT2 inhibitors, cardiac protection, autophagy, innate immunity

## Abstract

Sodium–glucose co-transporter 2 (SGLT2) inhibitors have been approved as a new class of anti-diabetic drugs for type 2 diabetes mellitus (T2DM). The SGLT2 inhibitors reduce glucose reabsorption through renal systems, thus improving glycemic control in all stages of diabetes mellitus, independent of insulin. This class of drugs has the advantages of no clinically relevant hypoglycemia and working in synergy when combined with currently available anti-diabetic drugs. While improving sugar level control in these patients, SGLT2 inhibitors also have the advantages of blood-pressure improvement and bodyweight reduction, with potential cardiac and renal protection. In randomized control trials for patients with diabetes, SGLT2 inhibitors not only improved cardiovascular and renal outcomes, but also hospitalization for heart failure, with this effect extending to those without diabetes mellitus. Recently, dynamic communication between autophagy and the innate immune system with Beclin 1-TLR9-SIRT3 complexes in response to SGLT2 inhibitors that may serve as a potential treatment strategy for heart failure was discovered. In this review, the background molecular pathways leading to the clinical benefits are examined in this new class of anti-diabetic drugs, the SGLT2 inhibitors.

## 1. Introduction

Patients with diabetes mellitus suffer from chronic morbidity with certain disabilities [1,2]. Anti-diabetic drug development has been one of the most important areas of medical research for this chronic disease affecting more than 463 million in the world [3]. The latest progress in the drug class development is sodium–glucose co-transporter 2 (SGLT2) inhibitors, which took an innovative approach to an age-old problem: to decrease glucose load in patients with diabetes. The SGLT2 inhibitors involve blocking glucose reabsorption in the proximal tubule of the kidney so that 90% of the sugar normally reabsorbed early in the proximal tubule is cleared in the urine. SGLT2 inhibition causes sodium to no longer be reabsorbed, and through natriuresis, additional metabolic benefits, including a reduction in body fluid, bodyweight, and blood pressure, as well as improved albuminuria, were observed [4]. Randomized clinical trials (RCTs) of SGLT2 inhibitors of empagliflozin, dapagliflozin, and canagliflozin showed that these drugs not only lowered glucose concentration in the serum, but also exerted cardiovascular (CV) benefits [5,6,7]. SGLT2 inhibitors also were shown to offer kidney protection in patients with diabetes [8] and improve heart failure (HF) with or without diabetes [9,10]. Regarding the improved hemodynamics by SGLT2 inhibition, there are hypotheses postulating that these drugs lead to both glucose and sodium reduction in the blood, resulting in an increase in urinary sodium excretion and reducing the reabsorption of both factors in the kidneys [11]. This effect reduces generalized congestion and intravascular volume, and decreases both cardiac afterload and preload, resulting in improved HF hospitalizations. Normally in a diabetic patient, the heart picks up the use of free fatty acids as a switch from glucose; however, this in turn impairs cardiac function [12]. The SGLT2 inhibitors, in this case, have been reported to increase ketone synthesis and ketones have been proposed to improve cardiac energetics and cardiac efficiency [13]. Ongoing research studies are conducted to shed more light on the relevance of the ketone hypothesis.

## 2. Clinical Trials

Many hypotheses have been proposed as to the potential mechanisms of benefit of SGLT2 inhibitors. The reduction in the risk of cardiovascular and renal adverse events with SGLT2 inhibitors occurs shortly after therapy initiation and persists with ongoing treatment. The speed of onset of the benefits strongly suggests that pleiotropic effects in addition to sugar-lowering are responsible, since it would take a respectable amount of time to achieve these benefits. Table 1 summarizes the RCTs that established the clinical benefits of the SGLT2 inhibitors. The Empagliflozin Cardiovascular Outcome Event (EMPA-REG OUTCOME) trial showed that in type 2 diabetes mellitus (T2DM), patients randomized to empagliflozin had cardiovascular benefit independently of baseline HbA1c levels and of reductions in HbA1c, including a significant 14% reduction of major adverse cardiovascular event (MACE), which is a composite of myocardial infarction, stroke, and cardiovascular death; a significant 38% reduction of cardiovascular death; a 32% reduction in all-cause death; and a 32% reduction of hospitalization for HF [5]. These results were followed by the Canagliflozin Cardiovascular Assessment Study (CANVAS) program, which also reported a significant 14% reduction of MACE in patients randomized to canagliflozin when compared with those on a placebo [6]. In the Dapagliflozin Effect on Cardiovascular Events-Thrombolysis in Myocardial Infarction 58 (DECLARE-TIMI 58) trial, reductions in hospitalization for HF were similarly observed in patients with HF with reduced ejection fraction (HFrEF) and HF with preserved ejection fraction (HFpEF) [7]. In the Canagliflozin and Renal Endpoints in Diabetes with Established Nephropathy Clinical Evaluation (CREDENCE) trial, the study was terminated early because primary endpoints were met earlier in patients randomized to canagliflozin by reducing the risk of the composite endpoint of end-stage kidney disease, doubling of serum creatinine, or renal or cardiovascular death by 30% [8]. Recently, in the Study to Evaluate the Effect of Dapagliflozin on the Incidence of Worsening Heart Failure or Cardiovascular Death in Patients with Chronic Heart Failure (DAPA-HF) trial, patients with HFrEF, regardless of the presence or absence of diabetes, randomized to dapagliflozin were associated with decreased hospitalization for HF [9]. In addition, in the Empagliflozin Outcome Trial in Patients with Chronic Heart Failure with Reduced Ejection Fraction (EMPEROR-Reduced) trial, among patients receiving recommended therapy for HF, those in the empagliflozin group had a lower risk of cardiovascular death or hospitalization for HF than those in the placebo group, regardless of the presence or absence of diabetes [10]. Table 1 summarizes the aforementioned trials.

## 3. Physiological Perspectives on Cardiac Protections

### 3.1. Glycemia

SGLT2 and, to a lesser extent, SGLT1 are responsible for tubular glucose reabsorption in proximal tubules and are integral to glucose homeostasis. The SGLT2 inhibitors block the reabsorption of sugar in the renal tubules independent of insulin action, and the increased urinary glucose excretion is associated with a lowering of fasting plasma glucose, postprandial glucose, and hemoglobin A1c (HbA1c). Thus, SGLT2 inhibitors decrease the chance of hypoglycemia that is frequently encountered in some other classes of anti-diabetic medications. Compared with sulfonylurea, SGLT2 inhibitors were observed with a 90% of risk reduction of hypoglycemia [14,15]. In addition, studies also have shown that SGLT2 inhibitors, in combination with complex insulin regimens, had lower insulin dose requirements and lowered body mass without incurring hypoglycemia [16]. RCTs showed that the three most commonly prescribed SGLT2 inhibitors (empagliflozin, dapagliflozin, and canagliflozin) are effective for a mean HbA1c reduction of 0.6% to 1.0% compared with placebo [5,6,7]. In patients with chronic kidney disease (eGFR 30–60 mL/min/1.73 m^2^), the HbA1c reduction was lessened [17]. The reduction of HbA1c was lowered to a certain extent depending on the combination therapy medication, which can be explained by the difference in the HbA1c reduction efficacy in different clinical trials.

### 3.2. Circulating Volume

Osmotic diuresis induced by SGLT2 inhibition, a distinctly different mechanism than that of other diuretic classes, results in greater electrolyte-free water clearance and fluid clearance from the interstitial fluid space than from the circulation that may relieve congestion, with minimal impact on blood volume [18]. In subjects receiving SGLT2i inhibitor, a twofold interstitial fluid volume reduction compared to blood volume was observed, while in subjects receiving diuretics, only a 78% reduction was seen [18]. Thus, by reducing the interstitial fluid volume to a greater extent than blood volume, SGLT2 inhibitors may provide better control of congestion without reducing arterial filling and perfusion. The natriuretic effect of SGLT2 inhibition is typically an increase in urine volume of 300 mL per day for the first 2–3 days that returns to baseline levels in several weeks, with re-establishment of the sodium–water balance and an approximately 7% reduction in plasma volume (interquartile range 5–12%) by 3 months of treatment [19].

Furosemide acts by inhibition of the luminal Na–K–Cl co-transporter in the thick ascending limb of the loop of Henle, causing sodium, chloride, potassium, and water loss in urine. Tolvaptan acts by inhibition of the V2 receptor, thereby decreasing the expression of the aquaporin channels, causing decreasing water reabsorption. Asymptomatic hyponatremic patients exhibit subtle disturbances in gait that improve following correction of the serum Na+ concentration [20,21]. A clinical study showed that the SGLT2 inhibitors are not merely a diuretic agent that generally uniformly reduces the body fluid volume regardless of the baseline fluid status, specifically the fluid response to dapagliflozin varies depending on the baseline volume fluid status [22]. Therefore, SGLT2 inhibitors exert different diuretic actions from loop diuretics and vasopressin V2 receptor antagonists and may induce a novel clinical benefit that may support the safety with low risk of fluid shortage when administering SGLT2 inhibitors to patients without fluid retention [22]. The diminished extracellular fluid reduction effect of SGLT2 inhibitors in patients without extracellular fluid retention may contribute to maintaining a suitable body-fluid status [22].

### 3.3. Body Mass

SGLT2 inhibitor use leads to increased excretion of glucose, resulting in an estimated loss of approximately 200–320 calories, corresponding to 50–80 g of urinary glucose per day [23,24]. Initially, it was thought that this may be solely responsible for the bodyweight loss caused by SGLT2 inhibitors, but other mechanisms for bodyweight loss were also suggested, because weight reductions were also observed even when administered to renal dysfunction patients with impaired urine secretion [25]. One possible mechanism was the effect of decreasing body-fluid volume due to the osmotic diuretic effect as a result of accelerated urinary glucose excretion, while another mechanism was suggested to be the promotion of fat decomposition [26]. Bioimpedance spectroscopy studies confirmed that the reduction in body mass with SGLT2 inhibitors was related to reducing both visceral and subcutaneous adipose tissue mass while preserving lean tissue mass [27,28]. SGLT2 inhibitor users typically report a bodyweight reduction of 2 kg in the first weeks of treatment that plateaus after 6 months, with around 3 kg weight loss [29,30]. In addition, at week 24, weight loss was positively correlated with anthropometric measurements (waist and hip circumferences) and hepatic enzyme levels, and negatively correlated with adiponectin, HDL-C, and β-Hydroxybutyrate levels [31].

### 3.4. Blood Pressure

Blood-pressure reduction by SGLT2 inhibitors is assumed to be related to their osmotic diuretic effect, although they have very slight natriuretic effects compared to diuretics. However, if osmotic diuresis was the sole mechanism, then the antihypertensive effect would diminish as kidney function deteriorates, but this is not the case [32]. A meta-analysis suggests that SGLT2 inhibition is consistently associated with systemic blood-pressure reduction of around 4 mmHg systolic and 2 mmHg diastolic [27]. The reduction in extracellular fluid volume seen with the initiation of SGLT2 inhibitor therapy is postulated to be the main reason for the initial systolic and diastolic blood pressure decrease of 5/2 mmHg in the first 2 weeks of therapy [33,34]. The decrease in systolic blood pressure at 1 month was accompanied by an increase in urinary volume and urinary excretion of both glucose and sodium, as well as bodyweight loss [35]. Over a longer course, a further reduction in body mass, owing to loss of visceral and subcutaneous adipose tissue, modulation of the RAAS, and reduced plasma uric acid levels, also is likely to contribute to a reduction in blood pressure [36,37]. The BP-lowering effect at 6 months after the administration of SGLT2 inhibitors is considered to be due to the plasma volume reduction resulting from both urinary sodium excretion and improvement of vascular endothelial function secondary to bodyweight loss induced by SGLT2 inhibition [35].

### 3.5. Heart Rate

Clinical data regarding treatment in Japanese patients with T2DM showed that SGLT2 inhibitors significantly decreased heart rate in patients with high baseline HR levels (≥70/min before treatment) [38]. Despite the reduction in blood pressure and plasma volume, heart rate did not increase with the use of SGLT2 inhibitors, suggesting inhibition of cardiac sympathetic tone and/or an increased parasympathetic tone were at play [27]. An elevated resting heart rate is a risk factor for vascular complications in patients with and without T2DM. Recently, the beneficial effects of SGLT2 inhibitors on cardiovascular and renal events were reported in large-scale clinical trials, and their mechanisms and the effect of SGLT2 inhibitors and resting heart rate were explored. Higher resting heart rate and HR and adipose tissue insulin resistance (adipo-IR) levels at baseline were independently associated with a greater reduction in resting heart rate [39]. In salt-treated obese and metabolic syndrome rats, which develop hypertension with an abnormal circadian rhythm of blood pressure, a non-dipper type of hypertension, did not exhibit a circadian rhythm of sympathetic nervous activity [38]. Treatment with the SGLT2 inhibitor tofogliflozin significantly decreased blood pressure and normalized circadian rhythms without inducing compensatory changes in heart rate, which may be the source of its beneficial effects on cardiovascular outcome in high-risk patients with T2DM [40].

### 3.6. Adipose Tissue

Obesity is defined as abnormal or excessive fat accumulation that can be detrimental to health, and is associated with decreased sensitivity to leptin, resulting in an inability to detect satiety despite high energy stores and high levels of leptin [41,42]. One common HF associated with T2DM is obesity-related HF with a preserved ejection fraction (HFpEF). It has been postulated that the synthesis of leptin in this disorder leads to sodium retention and plasma volume expansion, as well as to cardiac and renal inflammation and fibrosis [43]. Remarkably, leptin-mediated neurohormonal activation appears to enhance the expression of SGLT2 in the renal tubules, and SGLT2 inhibitors’ natriuretic actions at multiple renal tubular sites can oppose the sodium retention produced by leptin [43]. In addition, SGLT2 inhibitors reduce the accumulation and inflammation of perivisceral adipose tissue, minimize the secretion of leptin, and diminish its paracrine actions on the heart and kidneys to promote fibrosis [43]. Since such fibrosis is fundamental to the impairment of cardiac distensibility and glomerular function that characterizes obesity-related HFpEF, SGLT2 inhibitors can contribute to reduced perivisceral, perivascular, and pericardial adipose tissue deposition and inflammation. Furthermore, epicardial adipose tissue volume is associated with the severity of coronary artery disease and cardiometabolic disease, with resultant development of atrial fibrillation and cardiomyopathy [44].

In an animal study, empagliflozin increased energy expenditure, heat production, and the expression of uncoupling protein 1 in brown fat and in inguinal and epididymal white adipose tissue [45]. Furthermore, empagliflozin reduced M1-polarized macrophage accumulation while inducing the anti-inflammatory M2 phenotype of macrophages within white adipose tissue and liver, lowering plasma TNFα levels and attenuating obesity-related chronic inflammation [45]. Hence, empagliflozin suppressed weight gain by enhancing fat utilization and browning and attenuated obesity-induced inflammation and insulin resistance. Dapagliflozin improves glucose control, induces moderate weight loss, and reduces cardiovascular risk in patients with T2DM through decreasing epicardial adipose tissue by 20% from baseline [46].

### 3.7. Ketone Bodies

Recent large RCTs on SGLT2 inhibitors have demonstrated ample evidence of cardiac protection [5,6,7,8,9,10]. The proposed mechanism included SGLT2-inhibitors increasing circulating ketone bodies (such as β-hydroxybutyrate), with a subsequent increase in myocardial ketone body oxidation, serving to improve cardiac ATP production as a preferred substrate to fatty acids or glucose in the diabetic heart [47]. In addition, ketone bodies also bear a potent anti-inflammatory function that can reduce the atherosclerotic processes and slow the progression of cardiovascular disease [48]. Plasma levels of ketone bodies rise with prolonged treatment with SGLT2 inhibitors, and the shift toward ketone body production (and away from glucose oxidation) has been proposed as a potential mechanism for the rapid cardiovascular benefit of SGLT2 inhibitors [49]. In a mice study with empagliflozin, the presence of ketones, representing only a few percent of total calories from available fuels, did not increase cardiac efficiency, but rather resulted in an increased ATP production that in turn allowed improved bioenergetics of the heart [50].

### 3.8. Uric Acid

There is evidence that SGLT2 inhibitors can lower uric acid, which may also contribute to reduced cardio–renal risk. Uric acid is thought to increase oxidative stress and levels of reactive oxygen species, activate the renin–angiotensin–aldosterone system, increase levels of inflammatory cytokines, and induce activation of the NLRP3 (NACHT, LRR, and PYD domains-containing protein 3) inflammasome, seen in diabetic nephropathy and HF [51]. Studies have shown that uric acid promotes pro-inflammatory responses and fibrosis within the vascular wall, increases the turnover of vascular smooth muscle, increases the rate of apoptosis of endothelial cells, and depletes nitric oxide levels via reduced nitric oxide production and increased conversion into 6-aminouracil [52]. Chronically elevated circulating uric acid concentrations, therefore, are associated with increased risks of hypertension, cardiovascular disease, and renal disease [53]. Uric acid concentrations are often elevated in T2DM, and SGLT2 inhibitors can increase uric acid excretion, reduce circulating uric acid, and improve cardiovascular and renal function, leading to reduction of adverse cardiovascular events and slowing the progression of chronic kidney disease [54,55]. The mechanism by which SGLT2 inhibitors reduce serum uric acid has not been clearly established, but studies suggested that it may possibly involve the renal SLC2A9 (GLUT9) transporter [56]. The SLC2A9 gene encodes a facilitative glucose transporter and has two splice variants that are highly expressed in the apical membrane of the proximal tubule in the nephron, transporting both uric acid and d-glucose in the kidney [57]. The GLUT9, encoded by SLC2A9, which causes renal hypouricemia, is reportedly involved in SGLT2 inhibitor-mediated uric acid excretion via an increase in glucose excretion in the urine and an increased exchange of uric acid in the apical membrane of tubular cells [19,58]. In a meta-analysis of 62 clinical trials with SGLT2 inhibitors, uric acid was reduced by 35–45 μmol/L, with the effect seen within days of treatment initiation and persisting throughout the duration of the trial [59].

### 3.9. Endothelial and Vascular Functions

Several studies have reported improved endothelial function and reduced aortic stiffness with the use of SGLT2 inhibitors dapagliflozin and empagliflozin [60,61]. Within a few weeks of treatment with SGLT2 inhibitors, there was lowered office and 24 h ambulatory central systolic pressure that was determined by arterial stiffness of the large arteries. Since central systolic pressure is considered an important surrogate parameter of afterload relating to future cardiovascular outcomes, these data suggested the beneficial roles of SGLT2 inhibitors in patients with T2DM [62,63]. In addition, other parameters of arterial stiffness, such as central pulse pressure, forward pressure wave amplitude, and backward (or reflected) pulse wave amplitude, also showed significant improvement after SGLT2 inhibitor use [62,63] Taken together, the reductions in central blood pressure, pulse pressure, and forward wave amplitude in patients with T2DM can improve endothelial function and vascular stiffness, which subsequently decreases cardiovascular events.

### 3.10. Inflammation

The inflammatory reaction is currently thought to be the center stage of the development and progression of cardiovascular dysfunction, especially in patients with T2DM [64]. Through improving adipose tissue function and inducing decreases in serum leptin, cytokine, and chemokine concentrations, such TNF-α and IL-6, while increasing adiponectin, SGLT2 inhibitors serve to reduce inflammation [65]. Experimental findings showed that SGLT2 inhibition reduced circulating levels of C-C motif chemokine 2, IL-6, and TNF in *ApoE*^–/–^ knockout mice [66], and levels of TNF, IL-6, and C-reactive protein in hepatic cells and adipocytes in diet-induced obese mice [45]. Thus, the pleiotropic effects of SGLT2 inhibitors help to modify a host of inflammatory responses in a range of cells and tissues by multiple molecular pathways that improve oxidative stress, cytokine production, immune-system function, and obesity-related inflammation.

### 3.11. AGE-Mediated Effects

Advanced glycation end products (AGEs) and receptors for AGE (RAGE) play a role in diabetic cardiovascular disease. AGEs accumulate in the plasma and vascular tissues and, by directly interacting with the extracellular matrix, lead to arterial stiffness and decreased elasticity [67]. RAGE on the surface of endothelial cells, vascular smooth muscle cells, and monocytes promote oxidative stress, leading to inflammatory and fibrotic reactions in the cardiovascular system and arterial tree [68]. Activation of AGE/RAGE signaling is associated with an increased arterial wall stiffness, arrhythmias, coronary artery disease, acute myocardial infarction, systolic and diastolic dysfunction, and HF [69]. In a mice study, empagliflozin improved SGK1/ENaC profibrotic signaling and associated interstitial fibrosis, as well as left ventricular hypertrophy and left ventricular relaxation, in addition to glycemic indices [70]. In another diabetic rat study, treatment with empagliflozin also normalized endothelial function and reduced oxidative stress in the aorta with reversal of the pro-inflammatory phenotype and glucotoxicity (AGE/RAGE signaling) [71]. Methylglyoxal, a primary precursor of AGEs, decreases the phosphorylation of eNOSSer^1177^ and protein kinase B (Akt), resulting in inhibition of eNOS activity. SGLT2i may decrease the levels of methylglyoxal, preventing AGE formation and AGE/RAGE signaling, and thus improving decreased phosphorylation of eNOSSer^1177^ and Akt, consequently bestowing atheroprotective effects [72,73].

### 3.12. Autophagy

The relationship between autophagy and cardiovascular diseases is intricate. Although basal autophagy is critical to maintaining cell homeostasis, both increases and decreases in autophagy to an excessive degree can induce alterations in normal heart and blood vessel functions [74]. Autophagy is induced by stimuli such as hypoxia, oxidative stress, infection, endoplasmic reticulum stress, and nutrient starvation [73]. Dysregulation in autophagy is associated with a wide range of pathologies, including cardiovascular disease [74]. SGLT2 inhibitors were shown to suppress cardiomyocyte autosis (autophagic cell death) and confer cardioprotective effects in animal studies. Using myocardial infarction mouse models, treatment with empagliflozin significantly reduced infarct size and myocardial fibrosis, thus leading to improved cardiac function and survival [74]. During ischemia and nutritional glucose deprivation, when autosis is set in high gear, empagliflozin directly inhibits the activity of the Na^+^/H^+^ exchanger 1 (NHE1) in the cardiomyocytes to regulate excessive autophagy [75]. In primary isolated cardiomyocytes, empagliflozin was shown to improve myocyte contractility without affecting their beating frequency [75]. Since HF is associated with upregulation of NHE1 activity in the myocardium, with resultant increased cytosolic sodium and calcium concentrations in cardiomyocytes and increased oxidative stress and arrhythmogenesis, SGLT2 inhibitors may have therapeutic use [76].

### 3.13. Crosstalk between Autophagy and Innate Immunity

The autophagy core of the Beclin 1 protein complex moiety alters significantly after empagliflozin treatment [77]. In our dual epitope-tagging strategy to isolate Beclin 1 protein complexes, Beclin 1 was identified to bind with Toll-like receptor 9 (TLR9) and sirtuin 3 (SIRT3) after SGLT2 inhibitor treatment. TLR9 is an important receptor belonging to the innate immunity system. After binding to viral or bacterial DNA, TLR9 triggers pro-inflammatory reactions and activates innate immunity. SIRT3 is located in the mitochondria and has been implicated in regulating cellular respiration, ROS, and thermogenesis. The identification of the binding of Beclin 1–TLR9 and SIRT3 implies that SGLT2 inhibitors activate the communications between autophagy–Beclin 1 and innate immunity–TLR9 system.

The study showed that empagliflozin treatments enhance the activation of TLR9 and binding with Beclin 1 to trafficking to mitochondria and increase the abundance of SIRT3. The increased abundance of SIRT3 then direct the Beclin 1-TLR9 complex to increase the mitochondrial respiration rate and exert its protection against ROS and apoptosis. This Beclin 1–TLR9–SIRT3 complex triggers the communication of the autophagy and innate immune system and protects the mice from doxorubicin-related cardiac toxicity [77]. Empagliflozin’s protective effects are essentially abolished in the *SIRT3* or *TLR9* knockout mice. In patients with *SIRT3* point mutation and reduced enzymatic activity, the effects of empagliflozin on mitochondria is also reduced [77]. This evidence from mice to human signifies that the protective effects of SGLT2 inhibitors in the heart works through the collaborative effects of Beclin 1 in the autophagy machinery, TLR9 with innate immunity and inflammasome signaling, and the mitochondrial SIRT3.

The increased autophagy and activation of the Beclin 1/TLR9/SIRT3 axis lead to the question of whether other clinical effects of the SGLT2 inhibitors can be explained with this crosstalk. It is possible that there are tissue-specific functions of the Beclin 1–TLR9 complex. Moreover, the current SGLT2 inhibitors have certain degrees of SGLT1 inhibitions. It is not known whether SGLT1 inhibition plays any role in the Beclin 1–TLR9 axis.

**Figure 1:**
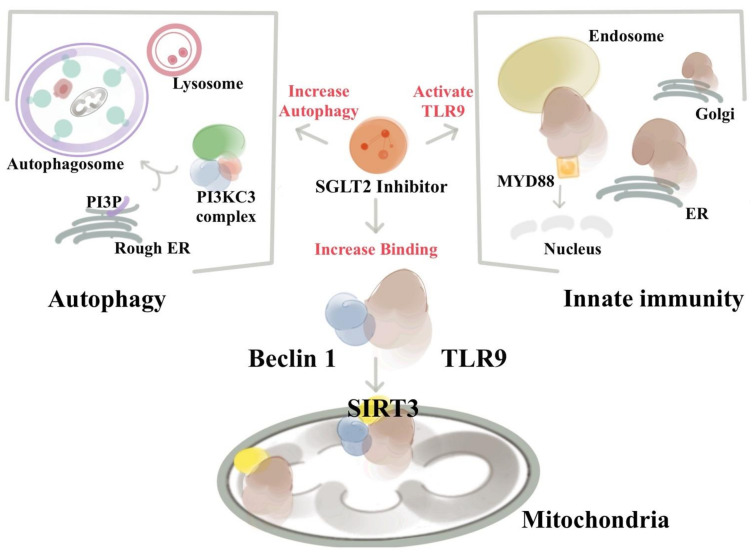
SGLT2 inhibitors activate the Beclin 1–TLR9–SIRT3 complex and connect the autophagy and the innate immune system. The existence of an integrated Beclin 1, TLR9, and SIRT3 network involving autophagy, oxidative stress, and mitochondria is essential for the ability of SGLT2 to protect the heart. SGLT2 inhibitor treatment enhances the activation of TLR9 to bind with Beclin 1 and increases the abundance of mitochondrial SIRT3. The bindings of Beclin 1 and TLR9 via SGLT2 inhibitors trigger the communications between the autophagic, innate immune system, and inflammatory machinery. The increased abundances of SIRT3 after SGLT2 inhibitor treatments then direct the Beclin 1–TLR9 complex to traffic toward the mitochondria, where the activated TLR9 enhances the mitochondrial respiration rate and exerts its protection against ROS and apoptosis. Of particular interest from a therapeutic standpoint is the finding that due to the deficiency of SIRT3 in mice and humans, both lose the SGLT2 inhibitors’ protective effects.

Recent findings also identified that TLR9 and Beclin 1 crosstalk to regulate muscle function and glucose metabolism during exercise [78], and along with the NAD-dependent deacetylase SIRT3, act in synergy in mitochondria in response to empagliflozin, and may serve as a potential treatment strategy for HF [77]. The possibility of an intersection between the autophagy machinery and innate immune responses indicates that bidirectional regulation between two systems may exist and provide a fine-tuning regulation with an SGLT2 inhibitor. TLR9 signaling is important in myocardial infarction, atherosclerosis, and cancer immunotherapy. The binding of Beclin 1 to TLR9 indicates that modulating Beclin 1 function with SGLT2 inhibitors may have therapeutic possibilities in these diseases (Figure 1).

## 4. Adverse Effects of SGLT2 Inhibitors

Using SGLT2 inhibitors in patients with T2DM is not, however, equivalent to taking a panacea. Certain side effects, namely genital tract infection, amputations, bone fractures, electrolyte imbalance, and importantly, diabetic ketoacidosis (DKA), have been reported in the large clinical trials [5,6,7]. While using SGLT2 inhibitors, DKA is observed occasionally in patients with T2DM with critical illness, or more often in patients with type 1 diabetes mellitus. The postulated mechanisms are: insulinopenia, expression of counterregulatory stress hormones, and increase in free fatty acids. A study of diabetic rats treated with SGLT2 inhibitors found the combination of insulinopenia and dehydration was the key to the development of DKA [79]. However, although euglycemic DKA in patients with T2DM using SGLT2 inhibitor has been reported, meta-analyses of clinical studies have found no increased risk of DKA for patients taking SGLT2 inhibitors compared to placebo [80].

## 5. Summary

**Figure 2:**
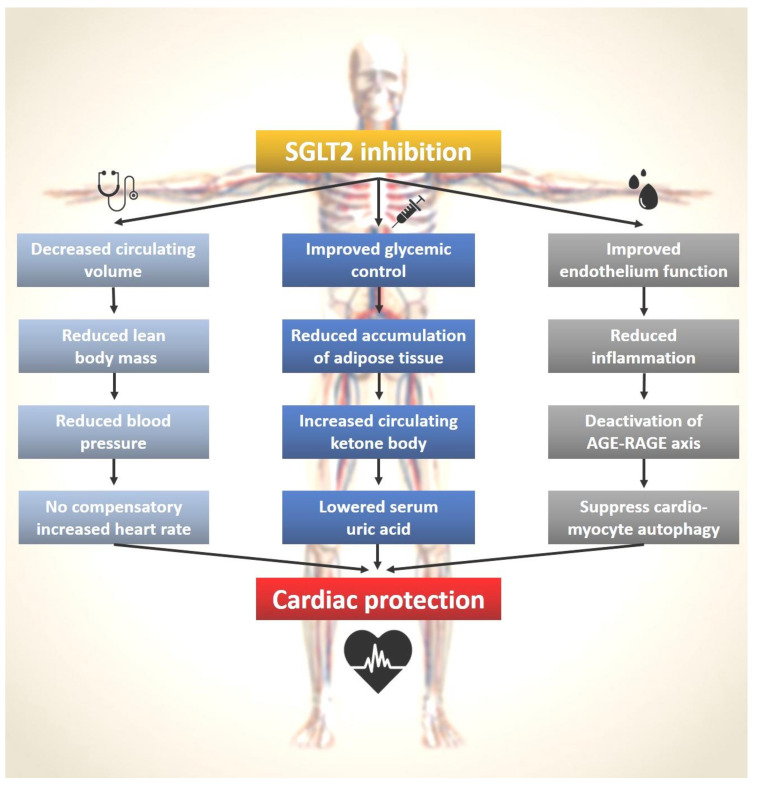
SGLT2 inhibitors and the pleiotropic effects on cardiac protection. The left panel with the stethoscope symbol shows the macroscopic actions of SGLT2 inhibitors that can be observe clinically. The middle panel with the syringe symbol illustrates the microscopic changes by SGLT2 inhibitors that can be checked via a blood test. The right panel with the symbol of a drop of blood outlines the molecular mechanisms of SGLT2 inhibitors that can be studied in basic science laboratories.

The pleiotropic effects of SGLT2 inhibition can be explained by the potential mechanisms through multiple pathways (Figure 2). The clinical benefits of SGLT2 inhibitors are observed clinically (left-side pathway), serum-level (central pathway), and molecularly (right-side pathway). In addition to the results from basic and translational research, SGLT2 inhibitors also did not fall short of the expected function to protect the heart. In our real-world study, we found that in patients with T2DM, SGLT2i as first-line treatment may be associated with decreased events of heart-failure hospitalization, acute coronary syndrome, and all-cause mortality, compared with metformin as first-line treatment [81].

## 6. Conclusions

SGLT2 inhibitors not only effectively decrease serum glucose, but also exert a variety of clinical benefits through decreased circulatory volume, reduction in systemic blood pressure, a shift towards ketone bodies as the metabolic substrate for the heart and improved endothelial function and suppression of AGE–RAGE signaling. Together, these pathways contributed to the cardiac protection shown in the clinical trials, including significantly improved cardiovascular events such as myocardial infarction, HF, and cardiovascular death. This consistent evidence of therapeutic benefits of SGLT2 inhibitors on a broad spectrum of cardiorenal endpoints in large RCTs should lead to the recommendation of these agents in updated ADA, AACE, ACC, AHA, and ESC guidelines as first-line treatments for atherosclerotic cardiovascular disease and HF.

## Figures and Tables

**Table 1:** Clinical Trials of SLGT2 Inhibitors.

	General Population			Special Population *		
Clinical Trials	EMPA-REG OUTCOME [5]	CANVAS Program [6]	DECLAR-TIMI 58 [7]	CREDENCE [8] (patients with CKD) *	DAPA-HF [9] (patients with HF) *	EMPEROR-Reduced [10] (patients with HF) *
Intervention	Empagliflozin 10 or 25 mg qd vs. placebo	Canagliflozin 100 or 300 mg qd vs. placebo	Dapagliflozin 10 mg qd vs. placebo	Canagliflozin 100 mg qd vs. placebo	Dapagliflozin 10 mg qd vs. placebo	Empagliflozin 10 mg qd vs. placebo
Population	7020 T2DM patients with 99.2% established CVD	10,142 T2DM patients with 65.6% established CVD and 34.4% CV risk factors	17,160 T2DM patients with 40.6% established CVD and 59.4% CV risk factors	4401 T2DM patients with 50.4% established CVD and 49.6% CV risk factors	4744 patients with 42% T2DM and 58% without T2DM	3730 patients with 50% T2DM and 50% without T2DM
Follow-up	3.1 years	3.6 years	4.2 years	2.6 years	1.5 years	1.3 years
Baseline A1c	7–10%	7–10.5%	6.5–12.0%	6.5–12.0%	No restriction	No restriction
eGFR	≥30	≥30	≥60	30–89	≥30	≥20
Primary outcomes	3P MACE, HR 0.86, 95% CI 0.74–0.99	3P MACE, HR 0.86, 95% CI 0.75–0.97	3P MACE, HR 0.93 95% CI 0.84–1.03; CV death or HHF, HR 0.83, 95% CI 0.73–0.95	New ESRD or 2x creatinine level or renal or CV death, HR 0.66, 95% CI 0.53–0.81	HHF, CV death, urgent hospital visit, and IV therapy for HF, HR 0.74, 95% CI 0.65–0.85	Adjudicated CV death or HHF, HR 0.75, 95% CI 0.65–0.86
CV death	HR 0.62, 95% CI 0.49–0.77	HR 0.87, 95% CI 0.72–1.06	HR 0.98, 95% CI 0.82–1.17	HR 0.78, 95% CI 0.61–1.00	HR 0.82, 95% CI 0.69–0.98	HR 0.92, 95% CI 0.75–1.12
All-cause mortality	HR 0.68, 95% CI 0.57–0.82	HR 0.87, 95% CI 0.74–1.01	HR 0.93, 95% CI 0.82–1.04	HR 0.83, 95% CI 0.68–1.02	HR 0.83, 95% CI 0.71–0.97	HR 0.92, 95% CI 0.77–1.10
MI	HR 1.18, 95% CI 0.70–1.09	HR 1.18, 95% CI 0.73–1.09	HR 1.18, 95% CI 0.77–1.01			
HHF	HR 0.65, 95% CI 0.50–0.85	HR 0.65, 95% CI 0.52–0.87	HR 0.65, 95% CI 0.61–0.88	HR 0.61, 95% CI 0.47–0.80	HR 0.70, 95% 0.59–0.83	HR 0.69, 95% CI 0.59–0.81

CI, confidence interval; CV, cardiovascular; CVD, cardiovascular disease; ESRD, end-stage renal disease; HHF, hospitalization for heart failure; HR, hazard ratio; IV, intravenous; MACE, major adverse cardiovascular event; MI, myocardial infarction; qd, once daily; T2DM, type 2 diabetes mellitus. * Patients with underlying chronic kidney disease (CKD) or heart failure (HF).

## Data Availability

Not applicable.
